# An optimized method of culturing neurons based on polyacrylamide gel

**DOI:** 10.52601/bpr.2023.230033

**Published:** 2024-02-29

**Authors:** Yongjing Qiao, Jihong Gong, Ziqi Jin, Yiting Tu, Xiaofei Yang

**Affiliations:** 1 Key Laboratory of Cognitive Science, Hubei Key Laboratory of Medical Information Analysis and Tumor Diagnosis & Treatment, Laboratory of Membrane Ion Channels and Medicine, College of Biomedical Engineering, South-Central Minzu University, Wuhan 430074, China

**Keywords:** Polyacrylamide gel, Neuron culture, Cell adhesion, Neural cell development

## Abstract

Substrate stiffness is a microenvironment with a certain stiffness constructed by the extracellular matrix and adjacent cells, which plays an important role in the growth and development of cells and tissue formation. Studies have indicated that the stiffness of the brain is about 0.1–1 kPa. The physiological and pathological processes of the nervous system are mediated by the substrate stiffness that the neurons suffer. However, how substrate stiffness regulates these processes remains to be studied. Culturing neurons on substrates with different stiffness *in vitro* is one of the best methods to study the role of stiffness in regulating neuronal development and activity. In this study, by changing the preparation time and the activation time of polyacrylamide gel, we provide an improved method that achieves a low toxic substrate environment for better primary neuron adhesion and development. Hope that this method is convenient for those studying the role of substrate stiffness in neurons.

## INTRODUCTION

Substrate stiffness is an elastic cellular microenvironment composed of an extracellular matrix and adjacent cells (Xia *et al.* 2020). Cells sense stiffness by detecting the surrounding environmental forces and responding to the environment through changes in the cytoskeleton (Lo *et al.* 2000; Rickel *et al.* 2020). Studies have shown that cells grown on softer substrates have a rounded shape, while cells grown on stiffer substrates have a flat shape (Gil-Redondo *et al.* 2022). Neurons are no exception, it has been demonstrated the ability of the cellular matrix to deform has a profound effect on the growth of neuronal cells and proper synaptic connections, while the mechanical properties of the substrate specifically guide the formation of neurite branches (Akiyama *et al.* 2014; Flanagan *et al.* 2002; Zhang *et al.* 2014).

Cells generate various responses to different substrate stiffness, such as the different ability of cell growth between the culture plates and softer hydrogels (Georges and Janmey 2005; Millar-Haskell and Gleghorn 2023). Therefore, simulation of the substrate of cell growth with different stiffness *in vitro* has become one of the important experimental methods to study the regulation law and mechanism of stiffness on cellular biological processes such as development and differentiation. At present, PDMS and polyacrylamide substrates with different stiffness have been widely reported for cell culture. PDMS is composed of different ratios of oligomeric base and curing agent Sylgard184, which can provide the substrate with a larger stiffness range, even up to ~MPa (Prager-Khoutorsky *et al.* 2011; Zhao *et al.* 2018). However, polyacrylamide gel (PA gel) can only prepare softer substrates with the range of 10 Pa to hundreds of kPa by varying the ratio of acrylamide to bis-acrylamide (Saha *et al.* 2010; Yi *et al.* 2022).

It has been reported that the tissue stiffness of the cerebral cortex is 0.1–1 kPa (Sun *et al.* 2012). However, how substrate stiffness regulates the growth and development of neurons in the cerebral cortex remains to be studied. PA gel can prepare a substrate that is close to the stiffness of brain tissue due to the particularity of its material (Teixeira *et al.* 2009). The culture conditions of primary neurons are harsher than those of tumor cells. In our previous experiment, we found that the traditional PA gel preparation method can be used to culture tumor cells or neurons for a few days. It was not so suitable for long-time culturing neurons to study synaptic formation, for the low survival rates and stunted growth.

Therefore, it is necessary to settle on a proper experimental method to prepare the substrate that cultured neurons. Through trial and error, we made step improvements to the traditional preparation method. By changing the preparation time and the activation time of PA gel, a simpler assay conducive to cell adhesion and the growth of primary neurons was achieved. The detailed preparation method is shown in the following.

## MATERIALS AND EQUIPMENT

### Materials and reagents

13-mm glass bottom dish (CELL E&G, GDB00001).

FBS (Invitrogen, 10099141); B-27 supplement (Gibco, 17504-010); L-Glutamine 200 mmol/L (Gibco, 25030-081); Neuronbasal (Gibco, 12800-017); 0.25% trypsin-EDTA phenol red (Gibco, 25200056); Poly-D-lysine hydrobromide (sigma, P6407); sulfo-SANPAN (CovaChem, 13414); Ammonium persulfate (APS; aladdin, A112450); N-[3-(Trimethoxysllyl)propyl]ethylenediamine (aladdin, T101385); Hoechst (Beyotime, C1028); 4% PFA (biosharp, BL539A); Alpha Tubulin Rabbit antibody (Beyotime, 11224-1); Acrylamide (aladdin, A108465-100g); Glutaraldehyde 25% aqueous solution (Ourchem, 111-30-8); 40% acrylamide stock solution (Sigma-Aldrich, cat. no. A4058); 2% bis-acrylamide stock solution (Sigma-Aldrich, cat. no. M1533).

### Animals

Postnatal day 0 (P0) pups of Kunming mice were used in this study. Mouse cortical neurons were obtained from the Kunming mice under the animal procedures performed in accordance with animal use rules and the requisite approvals of animal use committees of South-Central Minzu University.

### Equipment

Cell culture incubator (37 °C, 5% CO_2_), automated cell counter (RWD), inverted epifluorescence microscope (OLYMPUS), Laser confocal microscopy (NIKON).

## REAGENT SETUP

### Buffer solution

HEPES (100 mmol/L; Sigma, H4034) and NaHCO_3_ (4 mmol/L; Sigma, S8875) prepared in ddH_2_O with pH adjusted to 7.3–7.4 and osmotic pressure adjusted to 300–320 mOsm/kg, filtered with a 0.22 μm membrane, and stored at 4 °C in dark for up to one month.

Glutaraldehyde 0.5% aqueous solution prepared by glutaraldehyde 25% aqueous solution diluted in phosphate-buffered saline.

## PROCEDURE

### Prepare amino-silanated coverslip(s) (Fig. 1)

**Figure 1:**
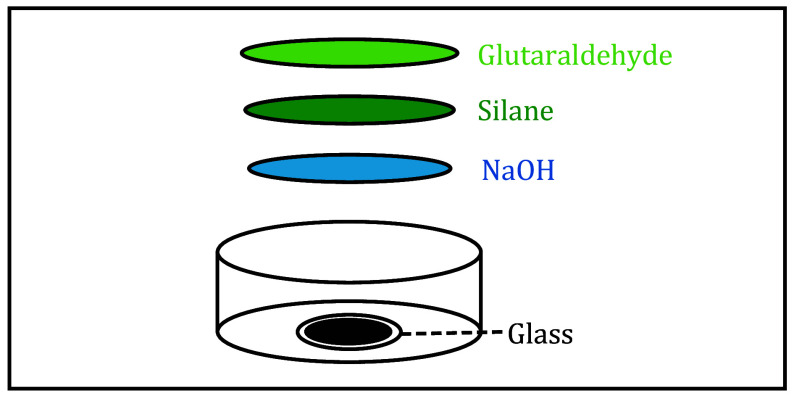
Diagram of amino-silicified coverslips. The preparation of amino-silicated coverslips including glass, NaOH, silane, and glutaraldehyde

(1) Add 200 μL of 0.1 mol/L NaOH to the center of the 13-mm glass bottom dish covering the entire glass surface and dry it naturally overnight.

A homogeneous NaOH film is important for the ability of the gel to be evenly distributed.

(2) Use a cotton swab to gently apply the silane onto a round slide and leave it for 6 min.

(3) Add ddH_2_O to wash the petri dish twice, each time about 10 min.

(4) Drain the ddH_2_O, add 100 μL of 0.5% glutaraldehyde, and allow it to react for 1 h.

(5) To remove 0.5% glutaraldehyde, add ddH_2_O to wash the petri dish for twice, each time about 10 min.

It is important that the unreacted 3-aminopropyltriethoxysilane need to be completely rinsed off to prevent it from forming a brownish-yellow precipitate with glutaraldehyde that fluoresces under ultraviolet light.

(6) Drain the ddH_2_O, activation step is completed.

**[CRITICAL STEP] **The amino-silanated coverslips remain viable for 48 h. However, it is best to use the amino-silanated coverslips immediately after they are created to ensure uniform gel attachment.

### Prepare polyacrylamide gel(s)

(7) Add ddH_2_O, 40% acrylamide and 2% bis-acrylamide to a 1.5 mL tube in turn, pipette and blow to mix, then add 10% ammonium persulphate and tetramethylene ethylene diamide (Table 1), blow gently to mix and add rapidly to the rounded areas of activated and drained Petri dishes. Add 15 μL of PA gel each to the activated and water-absorbed area of the Petri dish round glass slides.

**Table 1:** 0.6 kPa Configuration of polyacrylamide gel(s)

40% acrylamide	2% bis-acrylamide	ddH_2_O	APS	TEMED
75 μL	30 μL	895 μL	5 μL	0.5 μL

40% acrylamide and 2% bis-acrylamide should be mixed thoroughly by vortexing before use. After adding TEMED, to prevent solidification, mix and add to the petri dish immediately.

For the formulae of other stiffneFss substrates please refer to these documents (Tse and Engler^ 2010^).

(8) To make the gel congeal quickly, the petri dish can be placed in a 37 °C incubator.

It is necessary to place the Petri dish upside down to homogenizing the formed gel.

(9) When it has condensed, take the petri dish out of the incubator, add about 2 mL of 100 mmol/L HEPES, and lift the round coverslip with tweezers carefully.

(10) Remove the HEPES. Add HEPES and wash the petri dish again.

**[CRITICAL STEP] **It is best to proceed PA gel activation immediately.

### PA gel(s) activation (Fig. 2)

**Figure 2:**
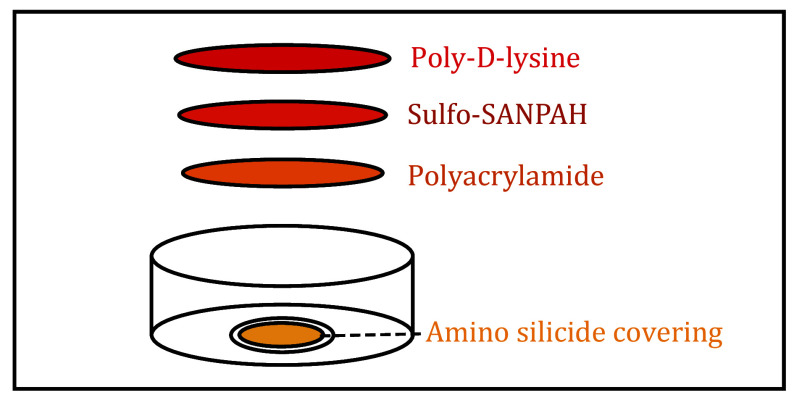
Schematic diagram of activating PA gel substrate. The reaction components of activating PA gel substrate consist of sulfo-SANPAN and poly-D-lysine

(11) Remove the HEPES from the petri dish, add 200 μL of sulfo-SANPAN, and irradiate with UV light for 6 min. The petri dish should be placed close to the UV light source that can be fully illuminated.

The gel was placed in the solution of Sulfo-SANPAH and covalently connected to the Sulfo-SANPAH to the polyacrylamide hydrogel under a violet external light source of 365 or 320 nm. N-hydroxysuperimide in sulfo-SANPAH can react with the primary amine of albumin to complete the adhesion of albumin to the gel surface. That is, polylysidic acid can be crosslinked to polyacrylamide gel for the adhesion of cells.

(12) After removing the SANPAN, add HEPES and wash the petri dish.

(13) Repeat the above procedure (Steps 11 and 12) once.

(14) Remove HEPES, add 200 μL Poly-D-lysine, and leave at 4 °C overnight.

Poly-D-lysine can also be changed to collagen or another amino-containing substance, such as fibronectin, if culture other types of cells, or other culture conditions require it.

### Dissociated cortical culture preparation

(15) Before the experiment, Poly-D-lysine in the PA gel dish needs to be removed and to PBS for later use.

(16) Prepare sterilized dissection instruments and dissection buffers.

(17) The cortical neurons of mice on the day of birth (p0) were isolated and taken to a 15 mL centrifuge tube with 1 mL of 0.25% trypsin-EDTA for 12 min in a 37 °C incubator.

(18) Remove the trypsin-EDTA. And add FBS to terminate digestion.

(19) Add the neuron cell culture medium to wash three times.

(20) Separate the cell tissue by cell sieve, and centrifuge the cell fluid at 300 *g* for 3 min. Cell counts were then performed after resuspension.

(21) Remove the PBS in the PA gel and add 25 μL of cell re-suspension.

(22) Add 1 mL of neuron culture fluid to the PA gel (day 0) after 1 h.

### Hoechst stained the cells and observed their adhesion

(23) Two hours later, add 10 μL of Hoechst to the medium for 10 min.

(24) Remove the culture medium and add 1 mL of PBS to wash it three times, each time about 5 min.

(25) Repeat the preceding steps (Steps 23 and 24) after 3 and 4 h.

(26) Observe the adhesion conditions of 2, 3 and 4 h by laser confocal microscope.

### Analyze neuron growth status by immunostaining

(27) After 24 h of culture, fix and stain the neuronal cells with the tubule skeleton protein Alpha Tubulin Rabbit antibody 1:500 dilution.

(28) Repeat it (Step 27) after 48 and 72 h.

(29) Observe the growth of day 1, day 2, and day 3 laser confocal microscopy.

## RESULTS

**Figure 3:**
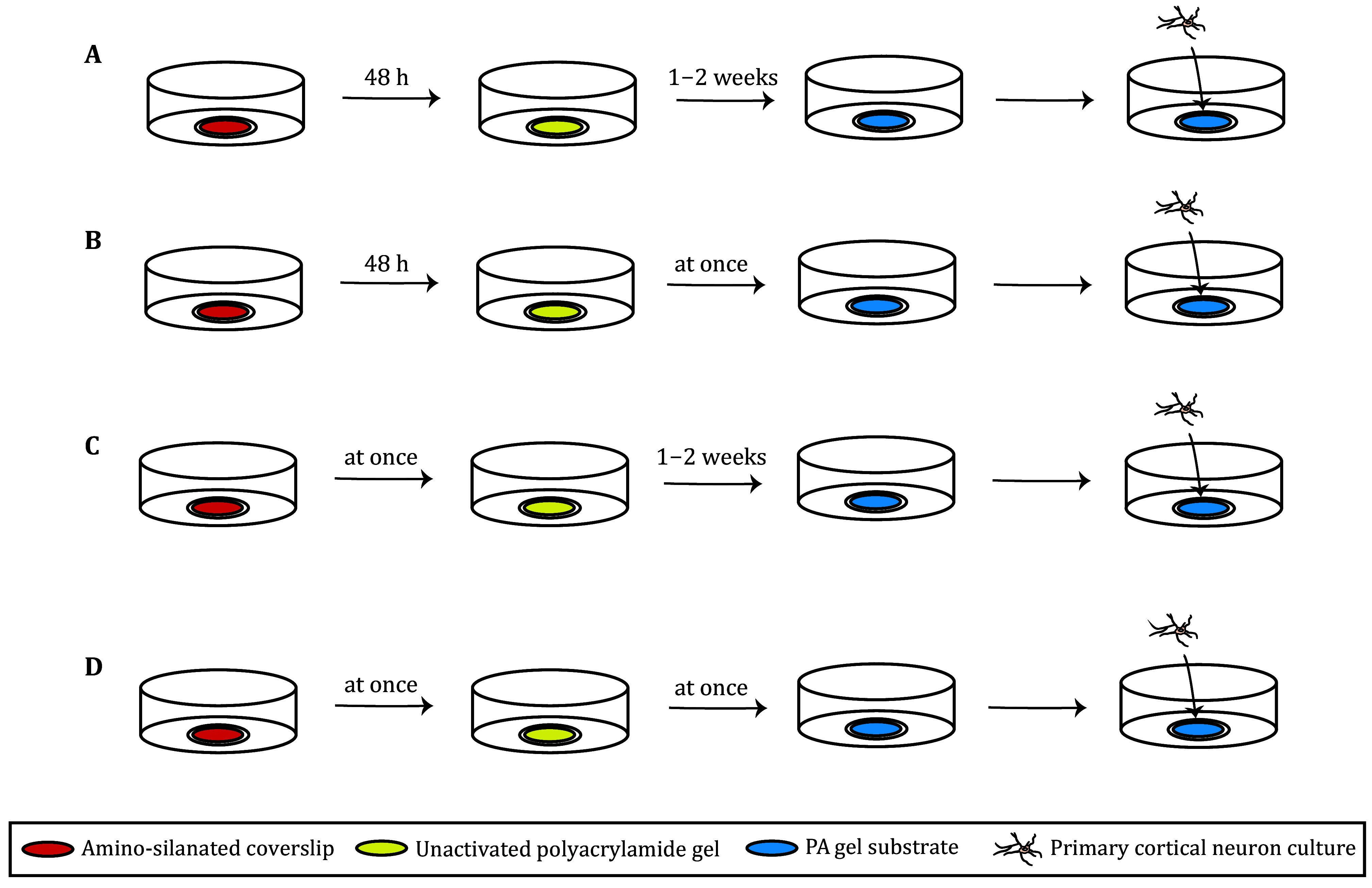
Schematic diagram of the optimized methods for preparing PA gel. **A** The original method of PA gel preparation. **B** Pre-improvement method I. **C** Pre-improvement method II. **D** The Optimized final method

In the process of preparing PA gel, both acrylamide and bis-acrylamide have certain toxicity. The preparation of the PA gel is usually divided into three steps: prepare amino-silanated coverslip(s), prepare PA gel(s), PA gel(s) activation, as described above. The first step is to activate the petri dish to prepare the amino-silicified covering, which can usually stay at this step for 48 hours. This is followed by the preparation of the PA gel. Then activation of the PA gel can be done after 1–2 weeks, sulfo-SANPAN is used for crosslinking the poly-D-lysine that adaptable for neuron adhesion. Due to the complexity of the gel preparation steps, in principle, the second and third steps can be prepared in large quantities at one time and stored for a period as shown in Fig. 3. In our previous study, we found this method was not suitable for culturing neurons, for the low survival rates and stunted growth. Especially to study the synaptic formation, the neurons need to be cultured in vitro for more than ten days, which is unrealized when using our previous PA gel preparation methods.

**Figure 4:**
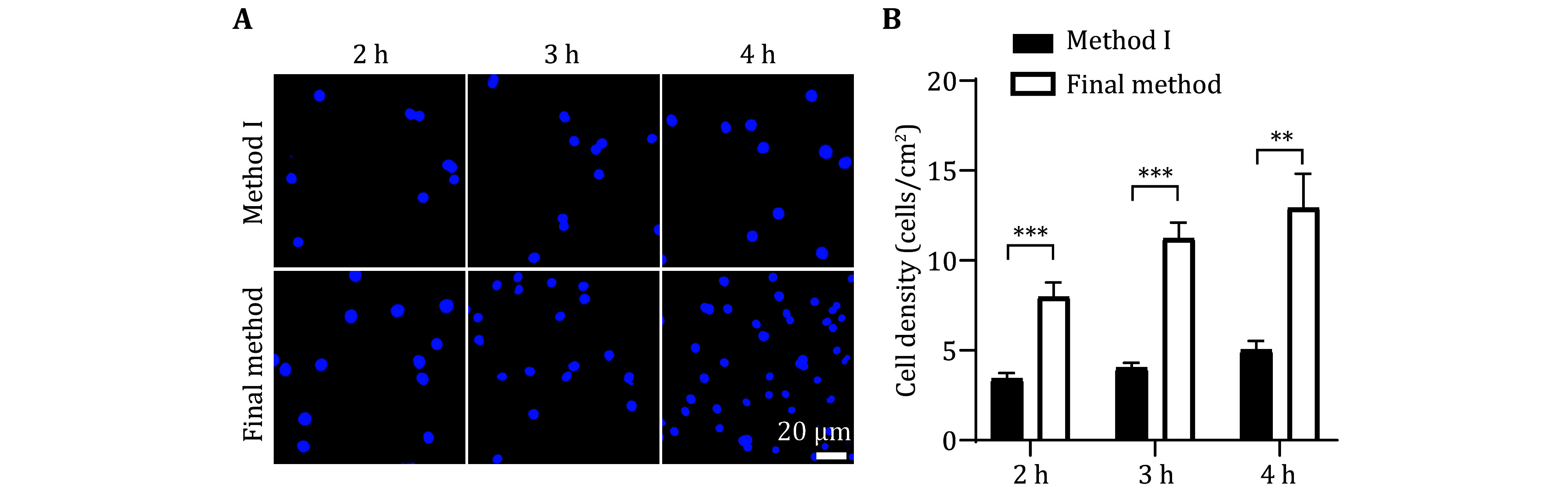
The optimized method (final method) improves the adhesion ability of neurons compared to method I. **A **Neurons cultured on the PA gel that were prepared by the final method and Method I independently. Representative images stained by Hoechst after neurons cultivating 2, 3, and 4 h. Scale bar, 20 μm. **B** Statistical graph of cell density from the neurons described in Panel A. At least 24 pictures from three independent cultures in each group. Data shows the average ± SEM; Statistical assessments were performed by *t-*test. **P < 0.01; ***P < 0.001

**Figure 5:**
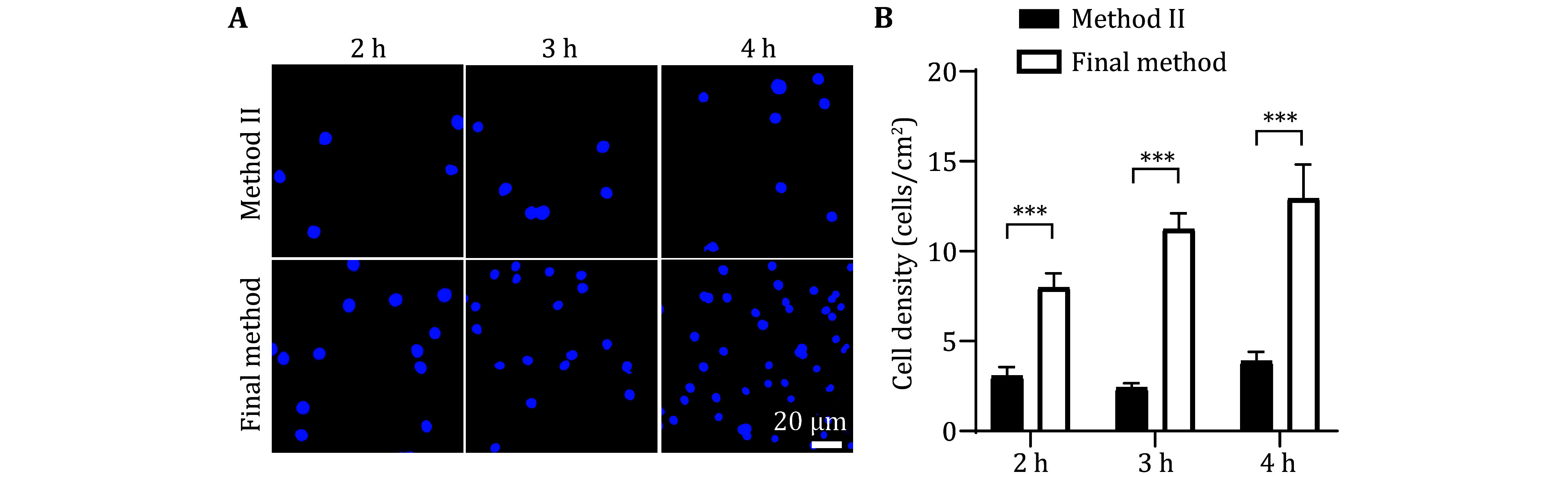
In the optimized method (final method), the neurons show stronger cell adhesion ability compared to Method II. **A** Neurons cultured on the PA gel that were prepared by the final method and Method II independently. Representative images stained by Hoechst after neurons cultivating 2, 3, and 4 h. Scale bar, 20 μm. **B** Statistical graph of cell density from the neurons described in Panel A. At least 24 pictures from three independent cultures in each group. Data shows the average ± SEM; Statistical assessments were performed by *t-*test. ***P < 0.001

**Figure 6:**
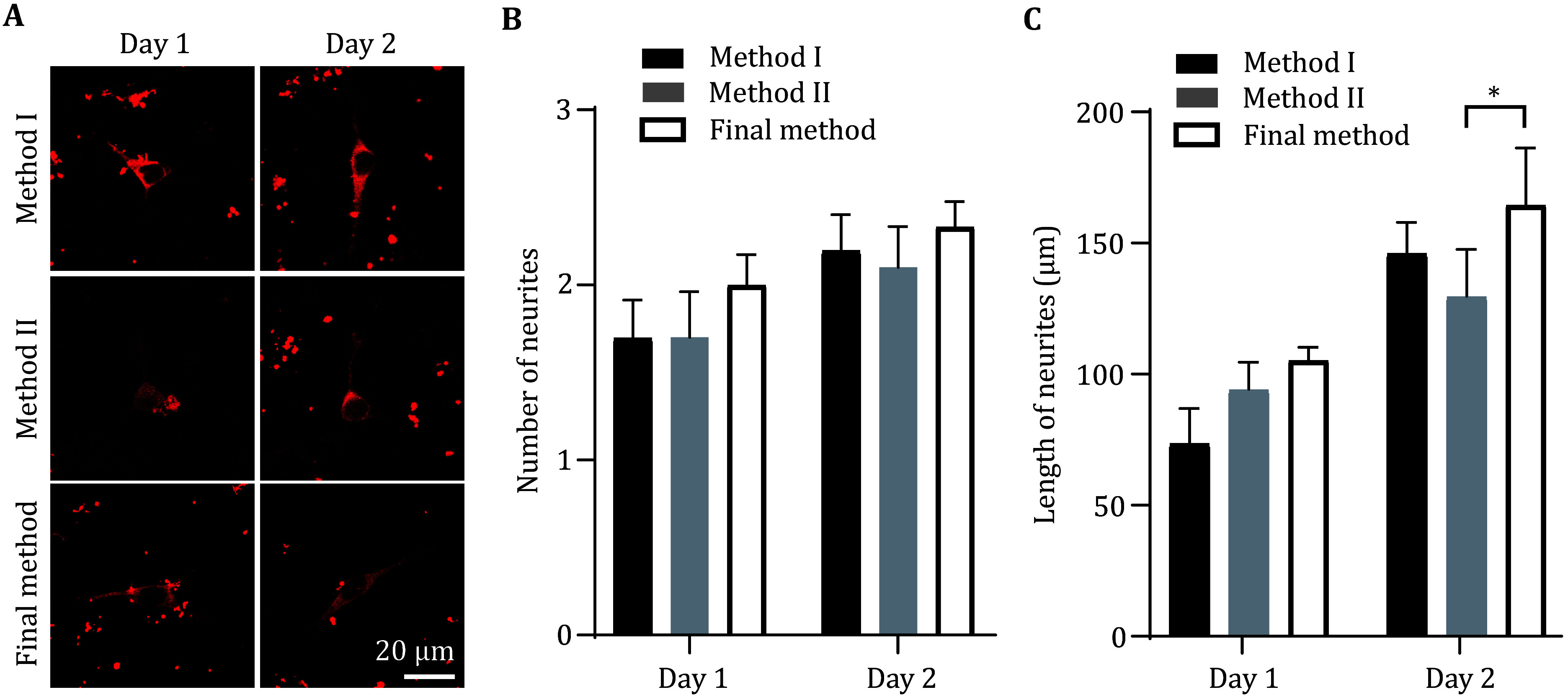
The neurons cultured by the optimized method (final method) show better development morphology compared to Method II. **A** Representative images obtained by immunostaining with Tubulin antibody after neurons cultivating one day or two days on PA gel were prepared by three different methods. Scale bar, 20 μm. **B**,**C** Statistical graph of neurite number (**B**) and neurite total length (**C**) from the neurons described in Panel A. At least 24 pictures from three independent cultures in each group. Data shows the average ± SEM; Statistical assessments were performed by *t*-test. *P < 0.05

Through experiments many times, we found that the neuron adhesion ability was significantly improved by modifying a few steps. Briefly, we improved the preparation process of PA gel from two aspects: making PA gel immediately after the preparation of the amino silicified covering and immediately activating the PA gel (Fig. 3). Then, we compared the adhesion rate (Figs. 4 and 5) and the growth status (Fig. 6) of neurons cultured on the PA gel with different improvement conditions.

In Fig. 4, we compared the ability of neuron adhesion between method I and the final method. The PA gel was simultaneously planted with 25 μL of primary neuron culture with a density of 4.62 × 10^5^/mL. Then the living cells were stained at 2, 3, and 4 h by using Hoechst after adhesion (Fig. 4). The data showed that the number of adhering neurons in the final method was significantly more than that in Method I. In Fig. 5, we compared the ability of neuron adhesion between Method II and the final method. The initial neuron implantation density and the living cell observation methods were the same as that in Fig. 4. As shown in Fig. 5, the number of adhering neurons in the final method was significantly more than that in Method II. The above data implied that the optimized final method is more conducive to the adhesion of primary neuron cells.

In addition, the PA gel prepared by the optimized final method, Method I and Method II were simultaneously planted with 25 μL of primary neuron culture with a density of 4.62 × 10^5^/mL. The cultured neurons for day 1, day 2, and day 3 were stained by tubulin antibody. The morphology and development of neurons were observed by using laser confocal microscopy (Fig. 6). Results showed that the total length of neurite has no difference in neurons of day 1 between the three methods. In neurons of day 2, the total length of neurites of the final method was significantly longer than method II. These results demonstrated that the optimized method is more suitable for neuron development.

In summary, our optimized final method not only increased the neuronal adhesion rate in the initial few hours after planted (Figs. 4 and 5), and exhibited better neuronal development after 2-days neuronal culture (Fig. 6). These evidences indicate that the improved method can greatly improve the survival rate of neurons on PA gel.

## DISCUSSION

The stiffness of PA gels can be controlled by adjusting the proportion of acrylamide and bis-acrylamide. It is often chosen to study the effect of substrate stiffness on cell growth and development for its controllability and low cost (Charrier *et al.* 2020; Saha *et al.* 2010). Substrate stiffness, as an elastic cell growth microenvironment, is currently considered to be an important factor affecting cell behavior (Wells 2008). However, the conditions for neuronal cultivation are harsh, especially for synaptic-formation studies, which require approximately two weeks of primary neuronal cultivation. It is difficult to culture neurons for that long time on PA gel. In this paper, we offered an optimized method of preparing PA gel: making PA gel immediately after the preparation of the amino silicified covering and immediately activating the PA gel (Fig. 3). Then, the cell adhesion and development of neurons were tested through experiments, and it was found that the optimized method significantly improved the cell adhesion rate and development of primary neurons (Figs. 4, 5, and 6). In the original method, it was left for a period after the reaction of glutaraldehyde and PA gel. For one thing, we speculate that the glutaraldehyde was easily oxidized to glutaric acid by oxygen due to its reducibility. However, glutaric acid could not react with the subsequent acrylamide, and these excess glutaric acids will generate toxicity to cell adhesion and growth in the later stage. For the other thing, one or two weeks were last after the preparation of PA gel with no activation, of which some molecules may have already failed in the HEPES buffer. The failed molecules could not react with sulfo-SANPAN in the crosslinking step. We have provided an optimized PA gel preparation method that effectively avoids the potential cytotoxicity caused by these defects. Also, we have achieved the cultivation of neurons on PA gel with different stiffness substrates for two weeks (data not shown), enabling them to form synapses effectively. Hope that this method offers convenience to researchers studying the role of substrate stiffness in neurons.

## Conflict of interest

Yongjing Qiao, Jihong Gong, Ziqi Jin, Yiting Tu and Xiaofei Yang declare that they have no conflict of interest.
